# Screening and purification of nanobodies from *E. coli* culture supernatants using the hemolysin secretion system

**DOI:** 10.1186/s12934-019-1094-0

**Published:** 2019-03-11

**Authors:** David Ruano-Gallego, Sofía Fraile, Carlos Gutierrez, Luis Ángel Fernández

**Affiliations:** 1 Department of Microbial Biotechnology, Centro Nacional de Biotecnología, Consejo Superior de Investigaciones Científicas (CSIC), Campus UAM-Cantoblanco, 28049 Madrid, Spain; 2 Research Institute of Biomedical and Health Sciences, Veterinary Faculty, Universidad de Las Palmas de Gran Canaria (UPGC), 35413 Arucas, Las Palmas, Canary Islands Spain

**Keywords:** *E. coli*/hemolysin, Nanobodies, Protein secretion, Single-domain antibodies

## Abstract

**Background:**

The hemolysin (Hly) secretion system of *E. coli* allows the one-step translocation of hemolysin A (HlyA) from the bacterial cytoplasm to the extracellular medium, without a periplasmic intermediate. In this work, we investigate whether the Hly secretion system of *E. coli* is competent to secrete a repertoire of functional single-domain VHH antibodies (nanobodies, Nbs), facilitating direct screening of VHH libraries and the purification of selected Nb from the extracellular medium.

**Results:**

We employed a phagemid library of VHHs obtained by immunization of a dromedary with three protein antigens from enterohemorrhagic *E. coli* (EHEC), namely, the extracellular secreted protein A (EspA), the extracellular C-terminal region of Intimin (Int280), and the translocated intimin receptor middle domain (TirM). VHH clones binding each antigen were enriched and amplified by biopanning, and subsequently fused to the C-terminal secretion signal of HlyA to be expressed and secreted in a *E. coli* strain carrying the Hly export machinery (HlyB, HlyD and TolC). Individual *E. coli* clones were grown and induced in 96-well microtiter plates, and the supernatants of the producing cultures directly used in ELISA for detection of Nbs binding EspA, Int280 and TirM. A set of Nb sequences specifically binding each of these antigens were identified, indicating that the Hly system is able to secrete a diversity of functional Nbs. We performed thiol alkylation assays demonstrating that Nbs are correctly oxidized upon secretion, forming disulphide bonds between cysteine pairs despite the absence of a periplasmic intermediate. In addition, we show that the secreted Nb-HlyA fusions can be directly purified from the supernatant of *E. coli* cultures, avoiding cell lysis and in a single affinity chromatography step.

**Conclusions:**

Our data demonstrate the Hly secretion system of *E. coli* can be used as an expression platform for screening and purification of Nb binders from VHH repertories.
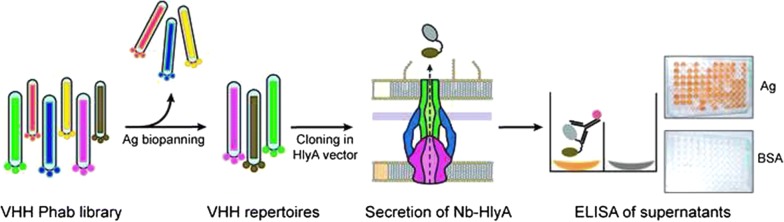

## Background

**Fig. 1:**
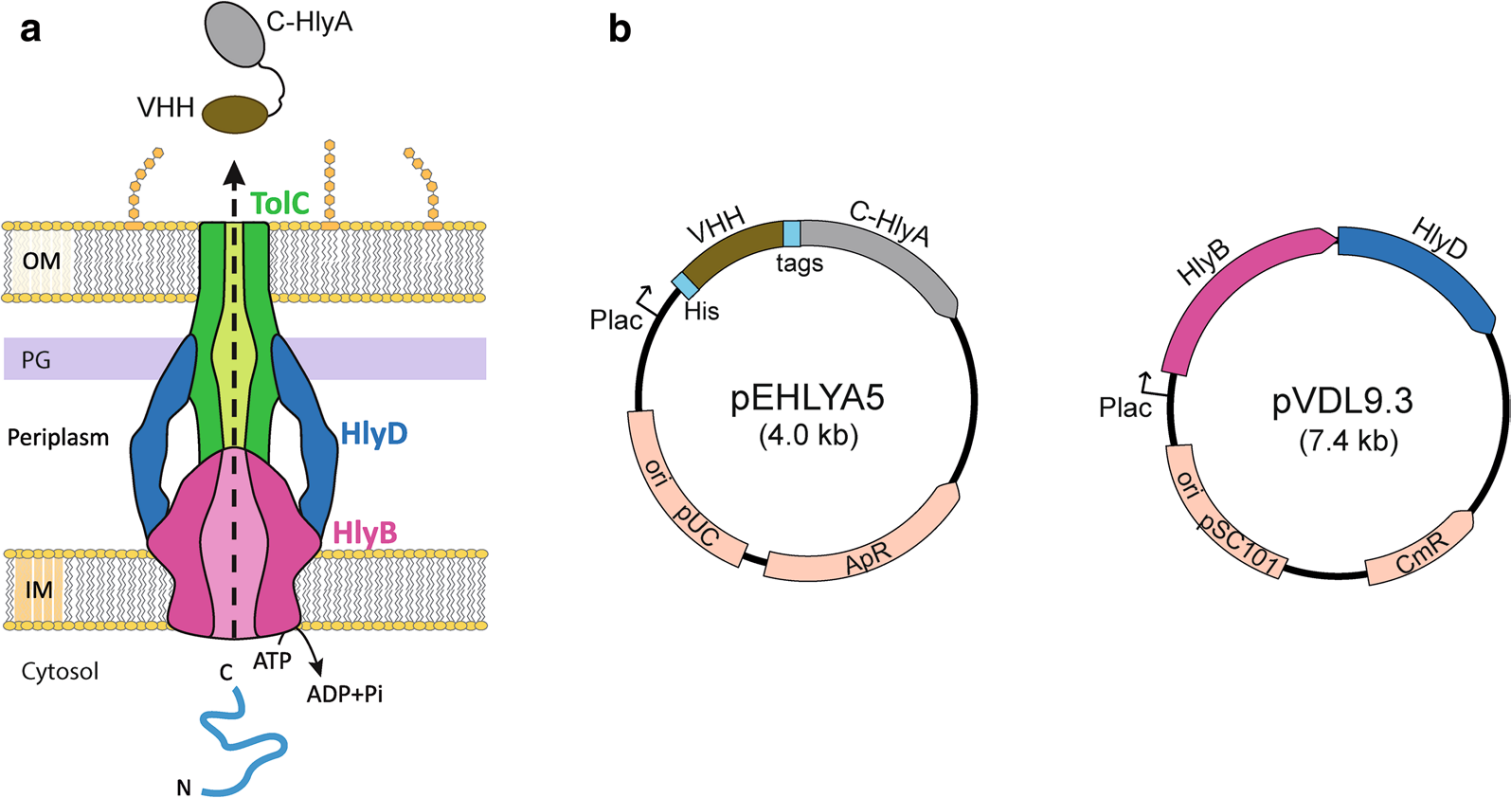
The *E. coli* hemolysin system for secretion of nanobodies. **a** Schematic representation of the HlyB, HlyD and TolC components of the Hly secretion system that spans the inner membrane (IM), the periplasmic space with the peptidoglycan (PG) layer, and outer membrane (OM) of *E. coli*. TolC is a trimeric OM protein with a large periplasmic domain; HlyB is an ATPase and forms a dimer in the IM. HlyD is a trimeric adaptor IM protein that interacts with HlyB and with the periplasmic domain of TolC. For simplicity, a longitudinal section of the Hly-protein complex is represented showing only two subunits of HlyD and a continuous open channel for protein export. The HlyBD complex recognises the C-terminal domain of HlyA (C-HlyA) in the bacterial cytosol to export the fusion protein with the nanobody (Nb) VHH domain to the extracellular medium. **b** Plasmids pEHLYA5 and pVDL9.3 used in this work for the secretion of Nb-HlyA fusions on *E. coli* bacteria (TolC+). Plasmid pEHLYA5 is used to generate fusion of the VHH sequence with an N-terminal His-tag and C-HlyA secretion signal. The linker region between the VHH and C-HlyA sequences includes tags for immunodetection (HA-tag, E-tag) and a human rhinovirus 3C protease recognition site. Plasmid pVDL9.3 encodes HlyB and HlyD components. Expression of the VHH-HlyA, HlyB and HlyD is controlled under the Plac promoter in both plasmids

Secretion of proteins to the extracellular medium reduces toxicity for the producing cell and eliminates the need of cell lysis for downstream processes [1, 2]. The hemolysin A (HlyA; 110 kDa) and the protein components required for its secretion were originally isolated from uropathogenic *E. coli* (UPEC) strains [3–6] and it serves as a paradigm of the bacterial Type I Secretion Systems (T1SS) [7, 8]. The Hly export machinery is composed of only three polypeptides, namely the inner membrane (IM) proteins HlyB and HlyD, and the outer membrane (OM) protein TolC. HlyB and HlyD are encoded, along with HlyA, in the Hly operon found in plasmids or the chromosome of UPEC strains, whereas TolC is encoded in a different location of the chromosome in most *E. coli* strains [4, 6, 9]. HlyB is a dimeric ABC-transporter anchored to the IM that has a cytosolic domain with ATPase activity [10, 11], while HlyD is a member of trimeric adaptor proteins [12] with a larger portion spanning much of the periplasm [13]. TolC forms homotrimeric OM channel comprising a β-barrel pore with long α-helical region that extends 10 nm toward the periplasm, forming a cylinder that opens to the extracellular medium, but is closed in the periplasmic entrance [14]. Upon engagement of HlyA in the cytosol by HlyB/D, TolC is recruited by this complex and its periplasmic entrance is opened to assemble a continuous channel connecting the IM and OM of *E. coli* [15–17] through which the HlyA polypeptide is secreted in a one-step mechanism, from the cytosol to the extracellular medium without a periplasmic intermediate (Fig. 1a) [7, 8]. TolC is a multifunctional protein that also participates in the secretion of other toxins and small molecules associated to different IM protein complexes [18].

Contrary to classical N-terminal signal peptides of proteins secreted to the periplasm by the Sec pathway [19], the signal for secretion of HlyA is located in the C-terminal end and is not removed during transport [20, 21]. The C-terminal location of HlyA signal also implies a post-translational mechanism of secretion. The last 50 amino acids of C-terminal end of HlyA appear to contain the signal recognized by HlyB/D complex [22, 23], but heterologous proteins have been secreted with this system fused to larger C-terminal fragments, comprising the last 60 amino acids [20, 24] and, more frequently, the last 218 residues (C-HlyA; 23 kDa), which includes three glycine- and aspartic-rich repeats, named as repeats in toxins (RTX) [7, 21]. Different heterologous proteins have been secreted in a functional form with C-HlyA, including the maltose binding protein [25], alkaline phosphatase [26, 27], β-lactamase [28], mammalian fatty acid binding protein [29], streptokinase [30], a single-chain Fv antibody [31], and a nanobody [32].

Nanobodies (Nbs) are recombinant single domain antibodies derived from the variable VHH domains from heavy chain-only antibodies (HCAbs) found in camelids (e.g., dromedaries, llamas, alpacas, etc.) [33, 34]. The VHHs have acquired important adaptations to be soluble and functional in the absence of the light chain, having longer and more diverse complementarity-determining regions (CDRs), strict monomeric behaviour, reversible folding properties, and higher resistance to proteolysis and thermal denaturation than VH domains from conventional antibodies [34]. These intrinsic biophysical properties facilitate their expression in bacteria, yeast, and mammalian hosts [35–38]. Their small size (ca. 14 kDa; 2–4 nm diameter) and long CDRs also allow binding of less accessible epitopes than those recognized by conventional antibodies, including active sites of enzymes [39, 40] and inner regions in the surface proteins of pathogens [41]. VHHs are also highly similar to human VH3 sequences, which is important to have a low immunogenicity in therapeutic applications of Nbs [42, 43]. These characteristics have made Nbs very attractive molecules for multiple applications, including cell biology studies [44], protein crystallography [45], therapy and in vivo diagnosis of human diseases [46–49]. Hence, methodologies for Nb selection, characterization and production are of great interest.

Secretion of Nbs to the extracellular medium of *E. coli* cultures could simplify the screening of clones binding an antigen of interest and their purification for in vitro characterization. Leakiness of the bacterial OM after overexpression in the periplasm allows, in some cases, the detection of Nbs in culture supernatants of *E. coli* [50, 51]. However, this is due to a non-specific release of periplasmic and cellular proteins due to bacterial lysis, and not to an actual secretion mechanism. Large accumulation of Nbs in the periplasm also leads to an impaired growth of the producing bacteria. Contrary to this, the Hly system specifically secretes the heterologous protein to the extracellular media, with little effect on the growth of the producing bacteria, given that the secreted protein is not accumulated in the periplasm and the integrity of the OM is not compromised [31]. Considering that a Nb binding α-amylase had been secreted in an active form with Hly-secretion system of *E. coli* [32], we wondered whether the Hly-secretion system could secrete a large diversity of Nbs that can be found in an immune library. With this aim, we constructed and screened with the Hly-system an immune library of VHHs against three protein antigens from enterohemorrhagic *E. coli* (EHEC), namely the extracellular secreted protein A (EspA), the extracellular 280 amino acid C-terminal region of Intimin (Int280), and the translocated intimin receptor middle domain (TirM) [52–54]. Our data show that screening of *E. coli* culture supernatants after secretion of Nb-HlyA fusions allows the identification of a diverse set of Nbs binding these antigens. We also demonstrate that the secreted Nb-HlyA fusions present disulphide bonds, indicating a correct oxidation state of the cysteine pairs. Lastly, secreted Nb-HlyA fusions were directly purified in a single affinity chromatography step from the supernatants of small-scale *E. coli* cultures (200 ml), with yields ranging between ca. 200–800 μg of purified Nb-HlyA fusion (~ 0.3–1.3 mg/l per OD600).

## Results and discussion

### Screening of Nbs against EspA, Int280, and TirM antigens of EHEC using the Hly secretion system

**Fig. 2:**
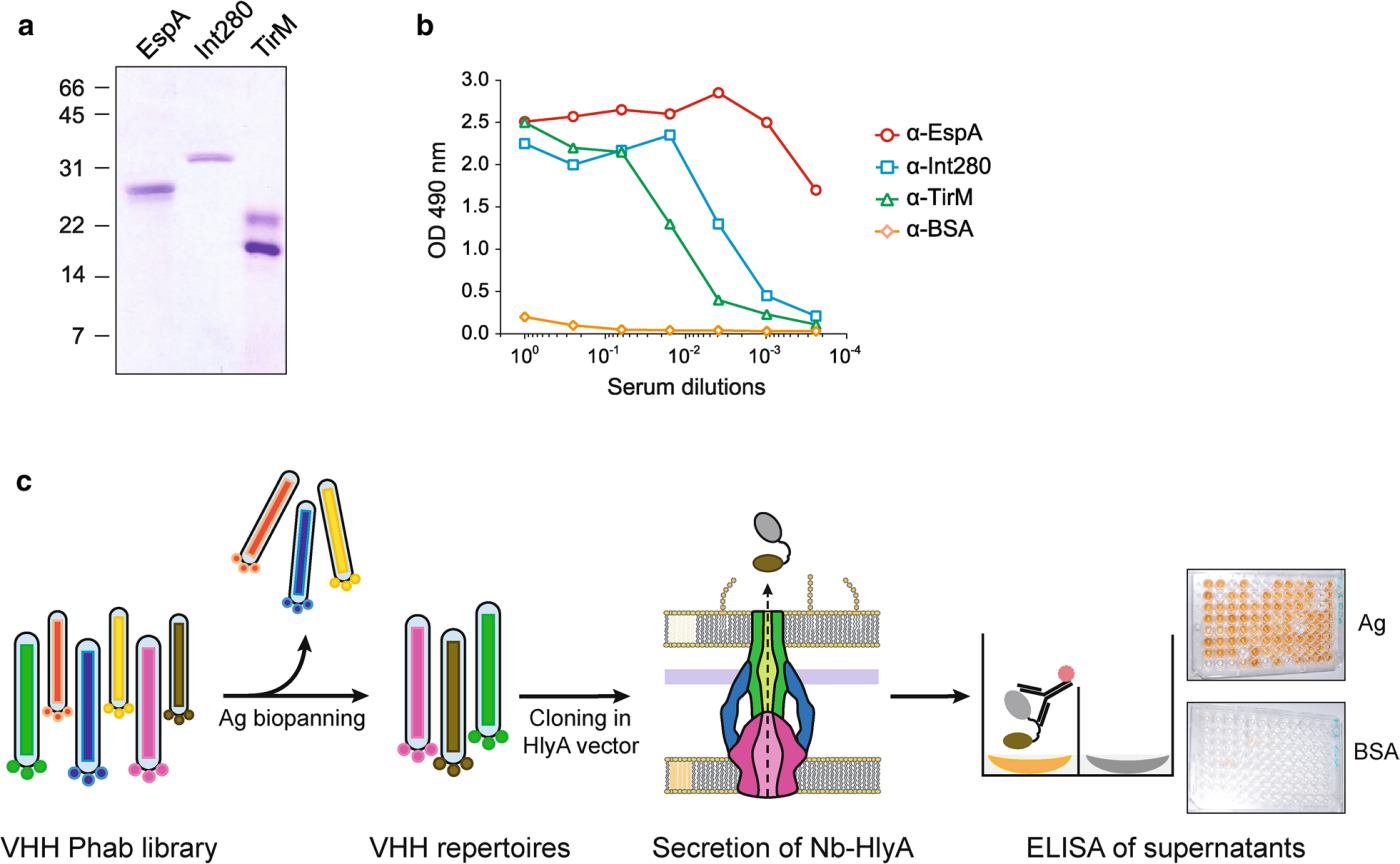
Identification of Nb binders secreted with the Hly-system from a VHH immune library against EHEC antigens. **a** Coomassie staining of purified EHEC protein antigens EspA, Int280 and TirM used for camel immunization. **b** ELISA of camel serum after immunization to reveal antibody response against EspA, Int280, TirM and BSA (negative control). The camel antibody response against each of the proteins using the indicated serum dilutions was developed with protein-A peroxidase (POD). **c** The VHH sequences amplified from the immunized animal were used to generate a phage antibody (Phab) library. Phab binders were enriched by panning to obtain VHH repertoires against each antigen (Ag). The VHH repertoires were cloned into pEHLYA5 and the Nb-HlyA fusions were secreted in *E. coli* bacteria carrying pVDL9.3. The culture supernatants of individual clones from each VHH repertoire were tested by ELISA against their corresponding antigen (EspA, Int280, TirM) and BSA (negative control). Bound Nb-HlyA fusions were developed with anti-E-tag-mAb

His-tagged versions of EspA, Int280, and TirM antigens from EHEC were purified and used for the immunization of a dromedary (*Camelus dromedarius*) (Fig. 2a). The generation of camel antibodies against these antigens in the blood serum of the immunized animal was confirmed by ELISA (Fig. 2b). The VHH gene segments were amplified by RT-PCR from the mRNA of peripheral blood lymphocytes (~ 2 × 10^7^) and cloned in a phagemid vector (pCANTAB 5Ehis) [31]. Upon transformation into *E. coli* strain TG1, a VHH phagemid library was constructed with a diversity of ≥ 1 × 10^7^ clones. Phage antibody (Phab) particles displaying the Nbs were produced and subjected to two cycles of biopanning in immunoplates coated with EspA, Int280, and TirM antigens (“Methods”). Phabs bound to each of the antigens were recovered, amplified in *E. coli* TG1 bacteria, and pool of phagemid DNA isolated for each repertoire.

**Fig. 3:**
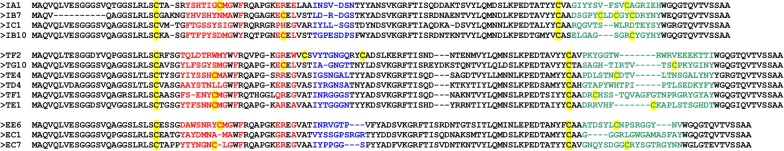
Amino acid sequence of VHHs clones identified after Hly-screening. Alignment of the amino acid sequences of the VHH domain in the clones selected by Hly-screening. The CDRs are labelled in colours: CDR1 (red), CDR2 (blue) and CDR3 (green). Cysteine residues are highlighted in yellow

To evaluate the capacity of the Hly system to secrete a diversity of functional Nbs, the three repertoires of VHHs were excised from phagemid DNA and fused to the C-HlyA signal in the vector pEHLYA5 (Fig. 1b). This multicopy plasmid (pUC18-derivative; Ap^R^) carries an IPTG-inducible Plac promoter, an N-terminal His-tag, unique *Sfi*I and *Not*I restriction sites flanking the VHH against α-amylase (Vamy) [32], a linker region having epitopes for immunodetection (HA- and E-tag) and the C-HlyA signal. The VHH repertoires were cloned replacing the Vamy sequence in pEHLYA5 and transformed into *E. coli* strain HB2151 (TolC+) harbouring pVDL9.3, which expresses HlyB and HylD under the control of Plac (Fig. 1b) [55]. 96 individual transformants from each repertoire were picked, inoculated in liquid media (96-well plates) and induced with IPTG. After centrifugation to remove bacteria, cultures supernatants were used directly in ELISA to determine binding to their respective antigen (EspA, Int280, or TirM) and to BSA, as a negative control antigen. To detect bound Nb-HlyA fusions, the ELISA plates were incubated with anti-E-tag mouse monoclonal antibody (mAb) and secondary anti-mouse IgG conjugated to peroxidase (POD). A scheme of the screening method is shown in Fig. 2c. We found that culture supernatants of ca. 30–70% of the clones, depending on the antigen, showed a positive ELISA signal against their corresponding antigen (OD490 ≥ 0.3), whereas their binding to BSA had signals ≤ 0.10. These data suggested the secretion of functional Nb-HlyA fusions against the three antigens with variable affinity and/or secretion levels. Between 24 and 40 positive clones for each antigen, showing diverse signals in the ELISA ranging between OD490 values 0.3 and 2.5 units, were chosen for plasmid preparation. The sequence of the cloned VHHs was determined and aligned for comparison, which confirmed the isolation of different Nb sequences with distinct CDRs for each antigen (Fig. 3). These clones were named after their recognized antigen (I for Int280, T for TirM and E for EspA). We found four different Nbs against Int280 (IA1, IB7, IB10, IC1), six Nbs against TirM (TD4, TE1, TE4, TF1, TF2, TG10) and three Nbs against EspA (EC1, EC7, EE6). Table 1 summarizes the identified Nbs, their frequency of isolation, and their CDR3 sequence.

**Table 1:** Nanobodies identified against EHEC antigens by Hly screening

Antigen	Representative clone	Frequency	CDR3 sequence
Int280	IA1	14/27	GIYYSVFSVCAGRIEH
	IB7	6/27	DSGPYCLDCGYCDRYNY
	IC1	6/27	RQGVTSWLRDTEYSY
	IB10	1/27	ELGAGSGRCYGYHY
TirM	TF2	10/24	PKYGGTWRWRVEEEKTTI
	TD4	4/24	SAGHTIRTVTSCPKYGINY
	TF1	4/24	PDLSTNCDTVLTNSGALYNY
	TG10	4/24	GTAPYWHTPIPTLSEDKYFY
	TE1	1/24	DRCHSSTQVAGFGTNPRGRYGYAY
	TE4	1/24	DRRVHFCKAPLSTSGHDT
EspA	EC7	33/40	ATDSYLCNPSRGGYNV
	EC1	6/40	GGGRLGWGAMASFAY
	EE6	1/40	GNQYSDGGCRYSGTRGYNN

**Fig. 4:**
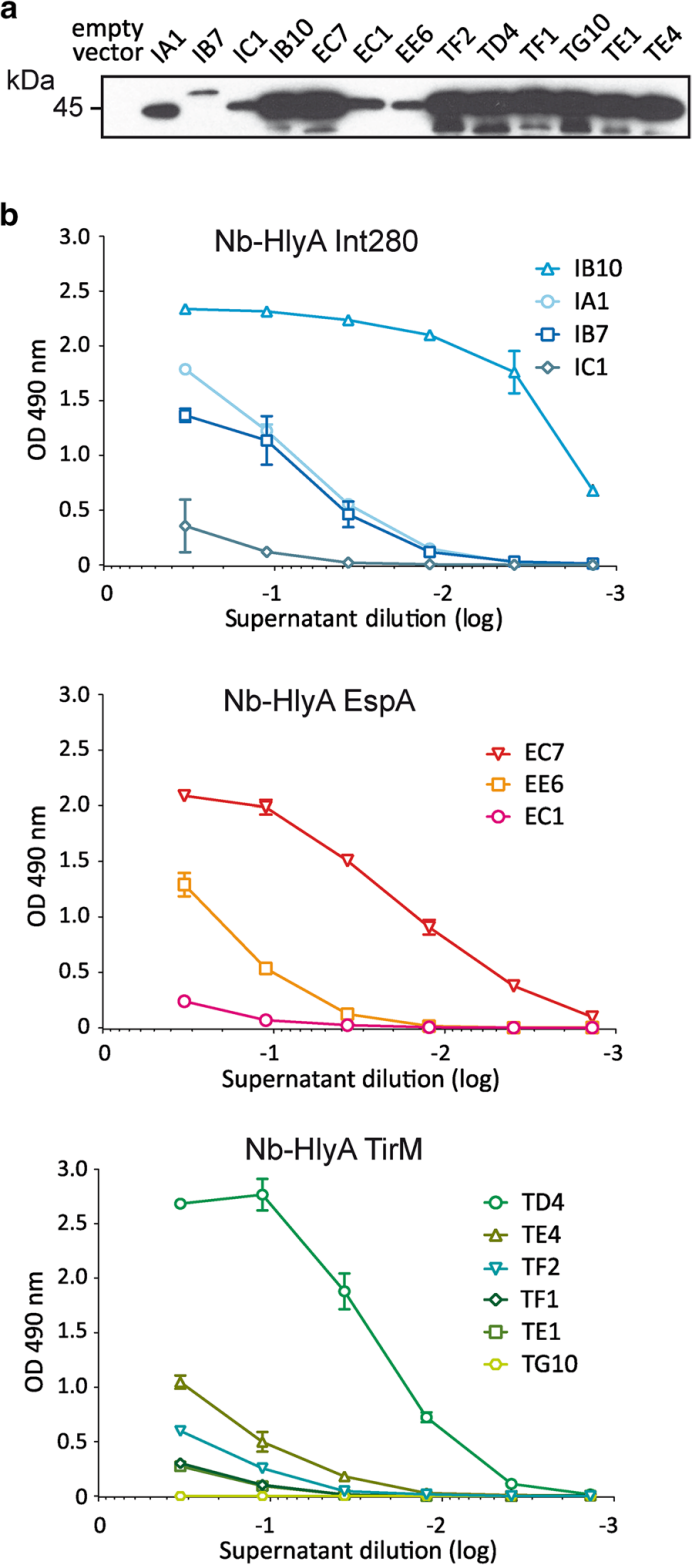
Secretion of Nb-HlyA fusions and antigen binding of representative clones. Representative clones of the identified VHH sequences against Int280, EspA and TirM were secreted as Nb-HlyA fusions. **a** Western blot developed with anti-E-tag-mAb of supernatants from induced cultures of the indicated clones showing a major protein band of ca. 42–45 kDa corresponding to Nb-HlyA fusions. The image shown is overexposed to visualize clearly protein bands of clones with lower expression levels. **b** ELISA of culture supernatants containing secreted Nb-HlyA fusions of the indicated clones against Int280 (top), EspA (middle) and TirM (bottom) developed with anti-E-tag mAb-POD. The represented OD490 values are the average from triplicate culture supernatants of each clone. Background signals to BSA are subtracted from the values obtained against the specific antigen (Int280, EspA, TirM)

Next, we compared the secretion levels and binding activity of representative clones from each group. *E. coli* bacteria carrying the empty vector (pUC18) were used as a negative control. Nb-HlyA fusions with the expected size (ca. 40–45 kDa) were detected by Western blot in the culture supernatant of all representative clones (Fig. 4a). The levels of secreted Nb-HlyA fusions were similarly high for 8 out of 14 clones, whereas the remaining 6 showed lower secretion levels. Binding of these clones to their respective antigen and BSA was compared by ELISA using serial dilutions of the culture supernatants. Results from these experiments are shown in Fig. 4b, representing the OD490 values obtained for the dilutions of the culture supernatant of each clone against the specific antigen, subtracting background values to BSA. From these data, clones IB10, TD4, and EC7 clearly showed the highest binding activities for their respective antigens, with positive ELISA signals at dilutions ≥ 10^−2^ (Fig. 4b). These results demonstrate that the Hly-system allows the secretion of different Nb sequences against all antigens tested, with variable expression levels and binding activities, as can be expected in the screening of VHH libraries. Hence, the Hly-system has the capacity to secrete a diversity of VHHs in a functional form, with secretion levels in the culture supernants high enough to allow the detection of positive Nb binders with different affinities for the antigen.

### Disulphide bonds in the secreted Nb-HlyA fusions

In *E. coli*, the cytosol is a strongly reducing environment, and cytoplasmic proteins usually do not form stable disulphide bonds [56, 57]. In contrast, cysteines in extracellular and periplasmic proteins secreted by the Sec-pathway are frequently oxidized, forming disulphide bonds catalysed by specialized Dsb-enzymes located, or having their catalytic sites, in the periplasm of *E. coli* [58, 59]. Proteins secreted by the Hly-system do not have access to the periplasm but, despite this, disulphide bonds were formed in a secreted scFv-HlyA fusion independently of Dsb-enzymes [60]. In contrast, when the same scFv was secreted fused to an autotransporter β-domain, which uses a periplasmic-dependent pathway, the presence of disulphide bonds required Dsb-enzymes (e.g. DsbA), which were not formed spontaneously in the extracellular medium [60].

Camelid VHHs frequently have, in addition to the conserved cysteine pair found in most immunoglobulin (Ig) domains, extra cysteine residues in the CDRs that also form disulphide bonds [61]. Out of the Nb sequences selected in the Hly-screening (Fig. 3), three clones have the two cysteines of the canonical disulphide bond of Ig-domains (IC1, TD4, EC1), whereas the rest contain one extra pair of cysteines (IA1, IB10, TF2, TG10, TE4, TF1, TE1, EE6, EE7) or two (IB7). We chose two clones having either two (TD4) or four (TF1) cysteines and investigated the oxidation state of the Nb-HlyA fusions, secreted and in the producing bacteria, by incubation with the thiol alkylating reagent 4-acetamido-4′-maleimidylstilbene-2,2′-disulphonic acid (AMS). AMS has a molecular mass of ca. 500 Da and its covalent binding to free thiol groups increase the protein mass and induce a retardation of its mobility in non-reducing SDS-PAGE. In contrast, proteins having disulphide bonds do not react with AMS and show a faster electrophoretic mobility.

**Fig. 5:**
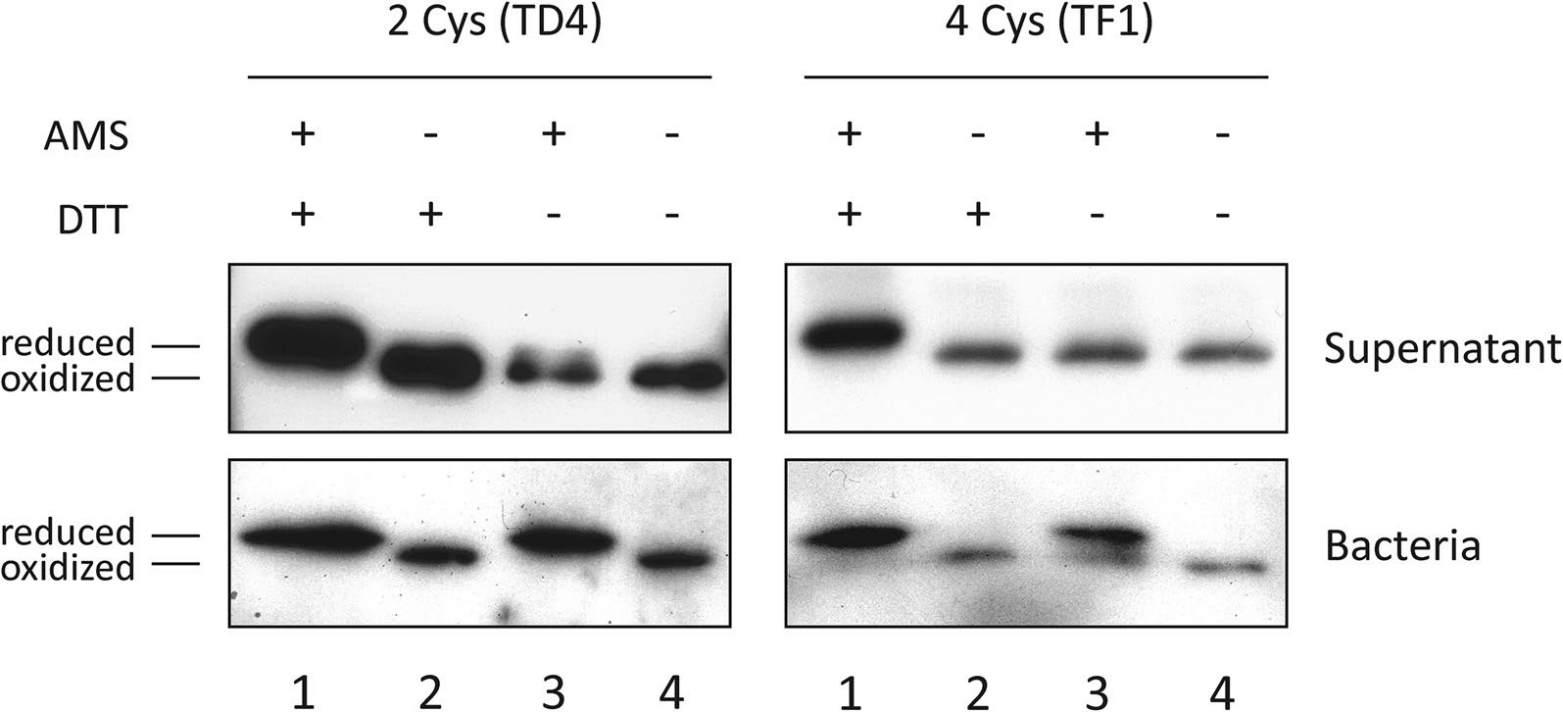
Disulphide bond formation in Nb-HlyA fusions. Thiol alkylation assay to determine the oxidation state of Cysteines (Cys) in secreted and cytoplasmic Nb-HlyA fusions from TD4 and TF1 clones, containing 2 and 4 Cys residues, respectively. Secreted proteins in culture supernatants and bacterial samples are treated (+) or not (−) with AMS (alkylating agent) and DTT (reducing agent), as indicated. Retarded mobility of alkylated polypeptides is indicative of reduced thiol groups. Protein bands of Nb-HlyA fusions developed after non-reducing SDS-PAGE and Western blot with anti-E-tag mAb

Bacterial cultures of TD4 and TF1 clones were induced and their culture supernatants and bacteria were collected. These samples were either directly precipitated with trichloroacetic acid (TCA), or previously incubated with dithiothreitol (DTT), to obtain positive controls of reduced samples before TCA-precipitation. Precipitated samples were resuspended in buffer having AMS, or lacking this alkylating reagent to obtain negative controls of alkylation (see “Methods”). All samples were subsequently subjected to non-reducing SDS-PAGE and Western blot. As expected, protein bands corresponding to Nb-HlyA fusions migrated differently depending on the treatment (Fig. 5). Secreted Nb-HlyA fusions were retarded in samples treated with DTT and AMS (reduced and alkylated controls; Fig. 5, lanes 1) and showed a faster mobility in untreated samples (Fig. 5, lanes 4), which were indicative of the mobilities of these Nb-HlyA fusions in their fully reduced and oxidized forms, respectively. Importantly, both Nb-HlyA fusions were found to be oxidized in the extracellular media treated with AMS (Fig. 5, lanes 3, Supernatants) and were reduced in the bacterial samples treated with AMS (Fig. 5, lanes 3, Bacteria). Non-alkylated samples had no migration drift. Hence, these data showed that thiol groups of Nb-HlyA fusions having 2 or 4 cysteine pairs, which are the most common among VHHs, have disulphide bonds in the secreted fusions but are reduced in the bacterial cytoplasm prior to their secretion. These results agree with those previously reporting the formation of disulphide bonds in a secreted scFv-HlyA fusion [60].

### Purification of Nb-HlyA fusions from culture supernatants

**Fig. 6:**
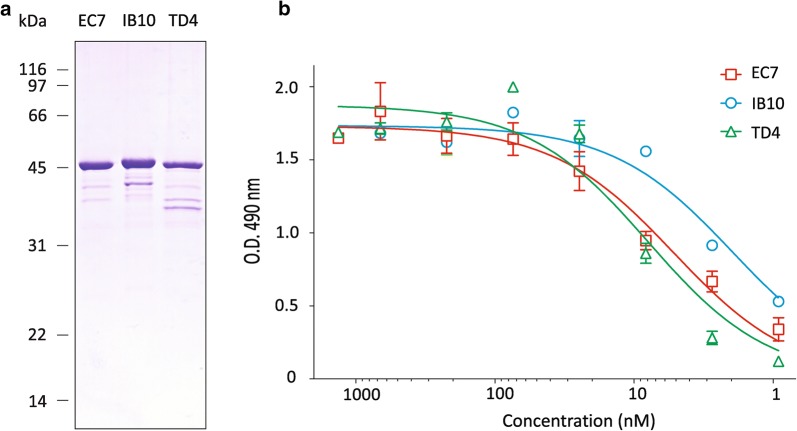
Purification and antigen binding activities of secreted Nb-HlyA fusions. **a** Coomassie staining after SDS-PAGE of purified His-tagged Nb-HlyA fusions from induced culture supernatants of EC7, IB10 and TD4 clones produced in *E. coli* HB2151(pVDL9.3). **b** Antigen binding curves determined by ELISA of purified Nb-HlyA fusions from EC7, IB10 and TD4 clones at the indicated concentrations. Bound Nb-HlyA proteins were developed with anti-E-tag mAb. OD490 values indicated are obtained against the specific antigen (EspA for EC7, Int280 for IB10, TirM for TD4) after subtraction of OD490 values against BSA (control antigen). Data are the average of triplicate ELISA experiments

To test the purification of secreted Nb-HlyA fusions, we chose the clones IB10, EC7 and TD4, which showed the higher binding signals against each antigen in culture supernatants (Fig. 3). *E. coli* HB2151(pVDL9.3) bacteria with the corresponding pEHLYA5-derivative were grown in 200 ml LB liquid cultures in shake flasks and induced with IPTG (see “Methods”). The culture supernatants were collected after centrifugation, equilibrated to PBS 1X and directly passed through a Cobalt-containing agarose resin for immobilized metal affinity chromatography (IMAC). The secreted Nb-HlyA fusions bound to the IMAC column were eluted by addition of imidazole. SDS-PAGE and Coomassie staining of the purified protein samples revealed major protein bands with the expected size of the Nb-HlyA fusions (ca. 42 kDa; Fig. 6a). Minor protein bands of smaller size are also visible, which likely correspond to proteolytic fragments of the full-length Nb-HlyA detected by Western blot (Fig. 4a). We quantified the amount of Nb-HlyA fusions obtained for each clone, which ranged between ca. 200–800 μg from these 200 ml cultures, indicating a yield equivalent to 0.3–1.3 mg/l per OD600.

Lastly, we confirmed the binding of the purified Nb-HlyA fusions for the corresponding antigen (i.e., Int280, TirM, EspA) by ELISA, using BSA as a negative control antigen. ELISA were developed with anti-E-tag mAb-POD and OD490 values to BSA (ca. ≤ 0.1) were subtracted from those obtained with the specific antigen at the different concentrations of the Nb-HlyA fusions (from 1 to 1000 nM; Fig. 6b). These data confirmed the activity of the purified Nb-HlyA fusions with curves indicating binding at low concentrations (1–10 nM). Hence, a single IMAC step allowed the purification of active Nb-HlyA fusions secreted in the culture supernatants of *E. coli*.

## Conclusions

The Hly-system had been used previously for the secretion of a model Nb binding α-amylase [32], but its potential to secrete repertoires of Nbs had not been tested. This work has shown that VHH repertoires can be secreted as Nb-HlyA fusions with the Hly-system of *E. coli*. Culture supernatants can be used directly in ELISA to identify antigen-specific binders among the secreted Nb-HlyA fusions, as we did for an immune library of VHHs against Int280, TirM and EspA antigens of EHEC. In addition, we have shown that active Nb-HlyA fusions can be purified from culture supernatants using a single IMAC step, avoiding bacterial cell lysis. Secretion facilitates purification, reduces toxicity for the producing bacteria and minimizes the action of intracellular proteases [1, 2]. Finally, we have demonstrated that the cysteine pairs found in VHHs form disulphide bonds in the secreted Nb-HlyA fusions. In contrast, these cysteines are reduced in the cytoplasm of bacteria, indicating that oxidation of Nb-HlyA fusions occurs associated to protein secretion. Taken together, these data reveal the Hly-system as a suitable platform for the secretion of Nbs in screenings of VHH repertoires, which could be enriched by phage display or alternative yeast and *E. coli* display technologies [36]. The amount of Nb produced in this work using small-scale shake-flask cultures is sufficient for in vitro characterization of the antigen-binding properties of the selected Nb clones. Higher yields of production may be attained in fed-batch cultures of *E. coli* (in bioreactors) in which very high cell densities can be achieved (OD600 > 50) and by optimization of the expression of Hly-export machinery and Nb-HlyA in the producing *E. coli* strain [1, 62–64].

## Methods

### Conditions for bacterial growth

*Escherichia coli* strains used in this work are listed in Table 2. Bacteria were grown at 30 °C or 37 °C, as indicated, in LB-agar plates (1.5% w/v) or in liquid LB medium. Antibiotics for plasmid selection were added at the following concentrations: ampicillin (Ap) 150 µg/ml; chloramphenicol (Cm) 30 µg/ml; kanamycin (Km) 50 µg/ml. Cultures of *E. coli* strain HB2151 carrying pVDL9.3 (*hlyB hlyD*) and the indicated pEHLYA5-derivative were grown overnight (o/n) at 30 °C (170 rpm) in liquid LB + Ap + Cm. Next, bacteria were inoculated in fresh medium and grown at 37 °C (170 rpm) until OD600 reached 0.5. At this point, bacteria were induced with 1 mM isopropyl-1-thio-β-d-galactoside (IPTG) and further incubated for 6 h with shaking (100 rpm). Average final OD600 determined for these cultures were ~ 3.0 (± 0.25). For secretion on 96-well plates, 200 μl of liquid media per well were used for growth and induction, as above. Culture supernatants were obtained after centrifugation (400*g*, 10 min). For secretion in flasks (200 ml of liquid media in 1 l flask), cultures were centrifuged twice (10 min, 10,000*g*, 4 °C) and the supernatants were collected for IMAC purification. In all cases, culture supernatants were equilibrated to PBS 1× (by adding 1/10th volume of PBS 10×) before downstream use.

**Table 2:** Bacterial strains and plasmids used in this work

Name	Relevant characteristics	Source or reference
DH10B-T1R	(F^−^ λ^−^) *mcrA* Δ*mrr*-*hsdRMS*-*mcrBC φ80lacZΔM15,* Δ*lacX74, recA1, endA1,* Δ(*ara, leu*)7697 *galU galK rpsL*(Str^R^), *nupG tonA*	Novagen-Merck
HB2151	Δ*lac*-*pro*, *ara*, *nal*^R^, *thi*, F’(*proAB lacI*^*Q*^ *lacZ*ΔM15)	[68]
TG1	Δ(*lac*-*proAB*) Δ(*mcrB*-*hsdSM*)5 (*rK*− *mK*−) *thi*-1 *supE* [F´ *traD36 proAB lacIqZ*Δ*M15*]	Stratagene-ThermoFisher
BL21 (DE3)	F^−^; *ompT hsdSB*(rB−, mB−) *gal dcm lon* λ(DE3[*lacI lacUV5*-*T7 gene1 ind1 sam7 nin5*])	Novagen-Merck
pCANTAB-5Ehis	Ap^R^; phagemid vector for periplasmic production of Nb-pIII fusions with His and E-tag	[31]
pEHlyA5	Ap^R^; pUC ori, *lac* promoter, N-terminal His tag, VHH, HA and E-tags, C-HlyA	This work
pVDL9.3	Cm^R^; expression of HlyB and HlyD	[55]
pET28a	Km^R^; pBR ori, T7 promoter, N-terminal His-tagged fusions	Novagen-Merck
pET28a-TirM_EHEC_	pET28a derivative; expression of His-tagged TirM of EHEC	This work
pET28a-Int280_EHEC_	pET28a derivative; expression of His-tagged Int280 of EHEC	This work
pET28a-EspA_EHEC_	pET28a derivative; expression His-tagged EspA of EHEC	This work

### Plasmids, DNA constructs, and oligonucleotides

Plasmids used in this study are listed in Table 2. *E. coli* strain DH10B-T1R was used as host for the cloning and propagation of plasmids. PCRs were performed with the Taq DNA polymerase (Roche, NZyTech) for standard amplifications in screenings and with the proofreading DNA polymerase Herculase II Fusion (Agilent Technologies) for cloning purposes. Int280 (residues 660 to 939 of Intimin), EspA (full-lenght) and TirM (residues 252 to 360 of Tir) sequences of EHEC were amplified by PCR from genomic DNA of EHEC strain EDL933*stx*- [65] and cloned *Eco*RI–*Hin*dIII into the pET28a plasmid backbone under the T7 promoter and fused to a N-terminal His-tag for purification. Plasmid pEHLY5 was constructed using vector pEHLYA2SD [31], by insertion of a chemically synthesized (GeneArt-ThermoFisher) *Xba*I–*Bam*HI DNA fragment of 566 bp encoding the His-tag-VHH(amylase)-HA-3Csite-E-tag region of pEHLYA5, which contains unique SfiI/NcoI and NotI sites flanking the VHH. All plasmid constructs were fully sequenced (Secugen, Madrid, Spain). Oligonucleotide used as primers (Sigma) are described in Table 3.

**Table 3:** Oligonucleotides used in this study

Name	Sequence (5′→3′)
CALL001	GTCCTGGCTCTCTTCTACAAGG
CALL002	GGTACGTGCTGTTGAACTGTTCC
VHH-SfiI2	GTCCTCGCAACTGCGGCCCAGCCGGCCATGGCTCAGGTGCAGCTGGTGGA
VHH-NotI2	GGACTAGTGCGGCCGCTGAGGAGACGGTGACCTGGGT
BamEcoTirM-EHEC	CGCGGATCCGAATTCCAGGCGCTTGCATTGACGCCGGAG
XhoHindInt280-EHEC	CCGCTCGAGAAGCTTTTACGATGAAACTTTCAGCTCCTCCTG
BamEcoInt280-EHEC	CGCGGATCCGAATTCATTACTGAGATTAAGGCTGATAAG
XhoHindInt280-EHEC	CCGCTCGAGAAGCTTTTATTCTACACAAACCGCATAGAC
BamEcoEspA-EHEC	CGCGGATCCGAATTCATGGATACATCAAATGCAACATCC
XhoHindEspA-EHEC	CCGCTCGAGAAGCTTTTATTTACCAAGGGATATTGCTGA

### Immunization and generation of the VHH library

Purified His-tagged Int280, TirM and EspA from EHEC were diluted in sterile water (2.5 ml) and mixed at 0.2 mg/ml to the same volume of adjuvant (Veterinary Vaccine Adjuvant, GERBU) reaching a total volume of 5 ml. This solution was injected subcutaneously to one male dromedary camel (*Camelus dromedarius*) corresponding to 0.5 mg of each antigen. After the initial immunization, four boosting immunizations were performed in the same manner once per week. Pre-immune serum was prepared from a small blood sample (5 ml) before the first injection. The immune serum was collected 7 days after the last immunization. Serial dilutions of pre-immune and immune sera were used in ELISA to confirm the immune response against the respective antigens with protein A-POD as secondary. Additional 50 ml of blood of the immunized animal were collected from the jugular vein in tubes containing EDTA as anticoagulant, using Venoject system. For lymphocyte isolation, 50 ml of RPMI-1640 medium (Sigma) were added to the 50 ml blood sample and the final mixture was divided in 4 aliquots of 25 ml. Each aliquot was added on top of a 25 ml of Ficoll-Paque Plus (StemCell Technologies) in sterile capped 50 ml Falcon tubes. After centrifugation (800*g*, 30 min, RT), lymphocytes were taken from the interphase, washed twice in RPMI-1640 by centrifugation (800*g*, 10 min), resuspended in 5 ml of RPMI-1640, and the number of cells determined in a Neubauer hematocytometer (Hausser Scientific). About 2 × 10^7^ cells were lysed with 2 ml of Trizol (Invitrogen) for RNA extraction following manufacturer instructions. The poly-A^+^ mRNA was purified from total RNA using an oligo-dT resin (Oligotex mRNA Minikit, Qiagen) and directly employed as template for first-strand cDNA synthesis reactions with random hexamers and oligo-dT primers (iScript cDNA Synthesis, Bio-Rad). About 1 μl of each cDNA synthesis was used as template in 50 μl PCR reactions with oligonucleotides CALL001 and CALL002. The amplified fragments of ≈ 0.6 kb, corresponding to VHH-C_H_2 domains, and ≈ 0.9 kb, corresponding to conventional V_H_-C_H_1-C_H_2 domains, were separated in 1.2% (w/v) low melting agarose gel and the ~ 0.6 kb band was purified (Qiaex II Gel Extraction kit, Qiagen). This fragment was used as template in a second PCR reaction with oligonucleotides VHH-Sfi2 and VHH-Not2 (Table 3) to finally obtain the amplified fragments of ~ 0.4 kb, corresponding to the VHH domains. The amplified VHH fragments were cloned *Sfi*I-*Not*I into the phagemid pCANTAB 5Ehis-backbone [66]. Ligations were electroporated in *E. coli* TG1 cells (Stratagene) and a library size of approx. 2 × 10^7^ clones were determined by plating dilutions on LB + Ap agar plates with 2% w/v glucose at 30 °C.

### Protein purification

Cultures of *E. coli* BL21(DE3) carrying the corresponding pET28a-derivative were grown at 30 °C in 500 ml of LB + Km and induced at OD600 ~ 0.5 with 1 mM IPTG during 2 h. Cells were harvested by centrifugation (10 min,10,000*g*, 4 °C), resuspended in 20 ml of Solution A—NaPO_4_ pH 7, 300 mM NaCl, DNase (0.1 mg/ml; Roche) and protease inhibitor cocktail (Roche)—, and lysed by passing three times through a French-Press at 1200 psi. The resultant lysate was ultracentrifuged (60 min, 40,000*g*, 4 °C) to obtain a cleared lysate supernatant. For purification of the His-tagged Int280_EHEC_, EspA_EHEC_, TirM_EHEC_, the lysates were passed through 2 ml of pre-equilibrated Cobalt-containing resin (Clontech) in a chromatography column and were washed with HEPES buffer (20 mM HEPES pH 7.4, 200 mM NaCl). The bound His-tagged proteins were eluted adding the same buffer complemented with 100 mM imidazole and were collected in 0.5 ml aliquots. For the purification of the secreted His-tagged Nb-HlyA fusions, supernatants from induced cultures were loaded at ca. 1 ml/min onto chromatography columns with pre-equilibrated Cobalt-containing resin (Clontech). Columns were washed with PBS and eluted with the same buffer containing 150 mM imidazole. Fractions eluted from metal-affinity chromatography were concentrated to a final ~ 1 ml in PBS using a 3 kDa centrifugal filter unit (Amicon Ultra-15, Millipore). Protein concentration was estimated using the Bicinchoninic acid protein assay kit (Thermo Scientific).

### Packaging of Phabs into M13 particles

For preparation of phage antibodies (Phabs) a mixture of *E. coli* TG1 cells representing the library or a subpopulation after panning, was incubated in 25 ml of LB-Ap supplemented with 2% (w/v) glucose at 30 °C until OD600 ~ 0.2. At this point, 10^10^ plaque forming units (PFU) of VCS-M13 helper phage (Km^R^; Stratagene) were added for 1 h incubation at 37 °C with gentle agitation. Then, *E. coli* cells were harvested by centrifugation (5 min, 4000*g*, RT) and resuspended in 250 ml of fresh LB + Ap + Km. After 16 h of incubation at 30 °C, the cultures were chilled on ice and centrifuged (10 min, 8000*g*, 4 °C). To recover the M13-particles from the supernatant, 50 ml of PEG-NaCl solution (20% w/v polyethylene glycol 8000; 2.5 M NaCl) were added and the resulting mixture was kept on ice for additional 45 min. Phage pellets obtained after centrifugation (20 min, 10,000*g*, 4 °C) were resuspended in 2 ml of TE (10 mM Tris–HCl, 1.0 mM EDTA, pH 8.0) and stored at − 80 °C.

### Library enrichment by Phab panning

All steps were performed at room temperature (RT). Each antigen (10 µg/ml, in PBS) was adsorbed for 2 h to 4 wells (50 µl/well) of a microtiter immunoplate (Maxisorb, Nunc). These solutions were discarded, and the wells were blocked by adding 200 µl/well of PBS with 3% w/v skimmed milk and 1% w/v BSA (Sigma). After 2 h, the blocking solution was replaced by a total of 2 × 10^11^ PFU of Phabs in 50 µl of PBS with 3% w/v skimmed milk. Phabs were allowed to bind for 1 h, and the unbound Phabs were removed from the plates by 20 washes of 1 min employing 200 µl/well of PBS with Tween20 0.05% and another 20 washes with PBS. To collect the bound Phabs, wells were incubated during 5 min with 0.1 M glycine pH 2.5 (50 µl/well). The solution from the wells was pooled together and immediately equilibrated by addition of one volume (400 µl) of 1 M Tris–HCl, pH 7.5. Phabs in this solution were later used to infect TG1, which were plated on LB + Ap agar plates. After 24 h incubation at 30 °C, colonies grown on these plates were harvested as a pool and used for phagemid packaging.

### Selection of individual specific binders

For rapid screening of individual Phab binders, a small-scale rescue of phagemids was performed in 150 µl cultures of TG1 grown in 96-well microtitre plates using 10^9^ PFU of helper-M13 phages. After production of the phagemids the plate was centrifuged (10 min, 600*g*, RT) and the supernatants (containing the Phabs) were used in ELISA to determine their specific binding to the antigen. At least 2 × 10^6^ independent colonies from each enriched Phab library were harvested from agar plates, and 50 units of OD600 were used for plasmid isolation (NucleoBond Xtra Midi, Macherey–Nagel). The VHH fragments were cloned *Sfi*I-*Not*I into the pEHLYA5 backbone vector under the Plac promoter. The size of each library was 2–3 × 10^6^ clones, as determined by plating dilutions on LB + Ap agar plates with 2% (w/v) glucose incubated at 30 °C. Isolated plasmids from the pools were transformed in strain HB2151 harbouring plasmid pVDL9.3 and individual colonies were isolated in 96-well microtitre plates with 200 µl of liquid LB-media with appropriated antibiotics and 1 mM IPTG for the secretion of the Nb-HlyA fusions.

### Alkylation of thiol groups in Nb-HlyA fusions

Protein samples from extracellular media and whole-cell bacteria were treated with the alkylating reagent 4-acetamido-4′-maleimidylstilbene-2,2′-disulphonic acid (AMS; ThermoFisher Scientific) as reported previously [60]. Briefly, for Nb-HlyA in supernatants, four aliquots of 800 µl from each induced culture supernatant were taken. Two of these aliquots were incubated with dithiothreitol (DTT, 25 mM) to obtain positive controls of reduced proteins. After incubation for 10 min at 37 °C, all four aliquots were precipitated with trichloroacetic acid (TCA, 10% w/v) for 1 h at 4 °C, and precipitated proteins were recovered by centrifugation (14,000*g*, 15 min). The protein pellets were washed twice with 1 ml of ice-cold acetone followed by centrifugation (14,000*g*, 15 min). Two of these protein pellets (AMS positive samples) were resuspended in 75 µl of a freshly prepared alkylation buffer (150 mM Tris HCl pH 7.5, 1% w/v SDS, 5% v/v glycerol, 15 mM AMS). The other two samples were resuspended in 75 µl of the same buffer without AMS. After 1 h incubation at 22 °C, 75 µl of non-reducing SDS-PAGE sample buffer was added (see below).

For intracelullar Nb-HlyA, bacteria from 1 ml of induced cultures were harvested, washed once, and resuspended in the same volume of LB. Samples were divided in four 200 µl aliquots and two of them were adjusted to 0.1 M DTT (DTT+ samples). Following 20 min incubation on ice, TCA was added (10% w/v). After 1 h incubation at 4 °C, the precipitated proteins collected by centrifugation (14,000*g*, 15 min, 4 °C). Pellets were washed once with 1 ml of ice-cold acetone, air-dried, and resuspended in 20 µl of alkylation buffer (AMS positive samples), or 20 µl of the same buffer without AMS. After incubation for 1 h at 22 °C, 10 µl of non-reducing SDS-PAGE sample buffer were added. All samples were boiled at 100 °C for 10 min before loading for SDS-PAGE (5–10 µl/well).

### SDS-PAGE and Western blot

Sodium Dodecyl Sulfate–Polyacrylamide gel electrophoresis (SDS-PAGE) and Western blot were performed following standard methods [67] using the Miniprotean III system (Bio-Rad). The sample buffer for reducing SDS-PAGE is 60 mM Tris–HCl pH 6.8, 1% w/v SDS, 5% v/v glycerol, 0.005% w/v bromophenol blue and 1% v/v 2-mercapto-ethanol (2-ME). In non-reducing SDS-PAGE, the sample buffer was devoid of 2-ME. Proteins separated by SDS-PAGE were either stained with Coomassie Blue R-250 (Bio-Rad) or subjected to Western blot. For the latter, the proteins were transferred to a polyvinylidene difluoride membrane (PVDF, Immobilon-P, Millipore) using semi-dry electrophoresis (Bio-Rad), as previously described [67]. Nb-HlyA fusions were detected with primary mAb anti-E-tag (Phadia, 1:5000) and secondary polyclonal rabbit anti-mouse IgG antibodies fused to POD (1:5000, Sigma). Membranes were developed using a mixture of 100 mM Tris–HCl (pH 8.0) containing 1.25 mM luminol (Sigma), 0.22 mM cumaric acid (Sigma), and 0.0075% (v/v) H_2_O_2_ (Sigma). The membranes were then developed by exposure to X-ray films (AGFA).

### Enzyme-linked immunosorbent assays (ELISAs)

ELISA conditions were based on those described previously [57]. Briefly, 96-well immunoplates (Maxisorp, Nunc) were coated for 120 min at RT with purified antigens (as indicated) diluted in PBS at a concentration of 5 µg/ml. Bovine serum albumin (BSA, Roche) was used as a negative control for detection. Phabs, culture supernatants or purified Nb-HlyA fusions were added to the wells at the indicated dilutions. For detection of bound Phabs, an anti-M13-HRP mAb (GE Healthcare) was added (1:5000). For detection of Nb-HlyA fusions, anti-E-tag mAb (1:2000; Phadia) and anti-mouse IgG-POD (1:2000; Sigma), as secondary antibody, were added. The reaction was developed with *o*-phenylenediamine (OPD, Sigma) and H_2_O_2_ (Sigma) and the OD490 was determined using a microplate reader (iMark ELISA plate reader, Bio-Rad).

## Abbreviations

Ab: antibody
ABC: ATP-binding cassette
AMS: 4-acetamido-4′-maleimidylstilbene-2,2′-disulphonic acid
Amy: α-amylase
Ap: ampicillin
BSA: bovine serum albumin
C-HlyA: C-terminal domain of 218 amino acids of HlyA
CDR: complementary determining region
Cm: chloramphenicol
Cys: cysteine
Dsb: disulphide bond
DTT: dithiothreitol
EHEC: enterohemorrhagic *E. coli*
ELISA: enzyme-linked immunosorbent assay
EspA: extracellular secreted protein A
HCAb: heavy-chain only antibodies
Hly: hemolysin
HlyA: hemolysin A
Ig: immunoglobulin
IM: inner membrane
IMAC: immobilized metal affinity chromatography
Int280: extracellular 280 amino acid C-terminal region of Intimin
IPTG: isopropyl-1-thio-β-galactoside
Km: kanamycin
mAb: monoclonal antibody
Nb-HlyA: nanobody fused to C-HlyA
Nb: nanobody
o/n: overnight
OD490: optical density at 490 nm
OD600: optical density at 600 nm
OM: outer membrane
OMP: outer membrane protein
OPD: *o*-phenylenediamine
PBS: phosphate-buffered saline
PFU: plaque forming units
PG: peptidoglycan
Phab: phage antibody
POD: peroxidase
PVDF: polyvinylidene difluoride membrane
RT-PCR: reverse transcription polymerase chain reaction
RT: room temperature
RTX: repeats in toxins
TCA: trichloroacetic acid
TirM: translocated intimin receptor middle domain
UPEC: uropathogenic *E. coli*
VHH: variable heavy chain domain from heavy-chain only antibodies
